# Spontaneous retroperitoneal bleeding secondary to celiac artery compression syndrome

**DOI:** 10.1002/ccr3.4158

**Published:** 2021-05-04

**Authors:** Kazuya Nagasaki, Hiroyuki Ariga, Toshiyuki Irie, Junya Kashimura, Hiroyuki Kobayashi

**Affiliations:** ^1^ Department of Internal Medicine Mito Kyodo General Hospital University of Tsukuba Mito Japan; ^2^ Department of Gastroenterology Mito Kyodo General Hospital University of Tsukuba Mito Japan; ^3^ Department of Radiology Mito Kyodo General Hospital University of Tsukuba Mito Japan

**Keywords:** celiac artery compression syndrome, posterior inferior pancreaticoduodenal artery dissection, retroperitoneal bleeding

## Abstract

Clinicians should consider celiac artery compression syndrome as the cause of ruptured visceral aneurysm and dissection and ask patients for unexplained chronic abdominal symptoms. Endovascular embolization with metallic coil placement is the first‐line treatment, and surgery can be avoided in some cases.

## Graphical abstract

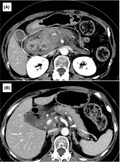

## 1 INTRODUCTION

A 56‐year‐old woman was found to have retroperitoneal hemorrhage secondary to isolated posterior inferior pancreaticoduodenal artery (PIPDA) dissection. She had chronic abdominal pain and celiac artery stenosis, suggesting that PIPDA dissection was associated with celiac artery compression syndrome (CACS). Clinicians may consider CACS as the cause of visceral dissection.

Celiac artery compression syndrome is a rare disorder characterized by chronic epigastric pain due to celiac artery compression by median arcuate ligament overgrowth.^1^ This syndrome is also known as median arcuate ligament syndrome or Dunbar syndrome. Its diagnosis requires confirmation of celiac artery stenosis, exclusion of other diseases, and characteristic symptoms. It is associated with visceral aneurysms and dissection, involving the celiac, mesenteric, and pancreaticoduodenal arteries.^2^, ^3^ The mortality rate for ruptured abdominal aneurysms is high, and early detection and treatment are critical.^4^ Herein, we report a case of a 56‐year‐old Japanese woman with sudden epigastric pain, who was diagnosed with acute retroperitoneal hemorrhage secondary to isolated PIPDA dissection, accompanied by CACS.

## 2 CASE HISTORY/EXAMINATION

**FIGURE 1:**
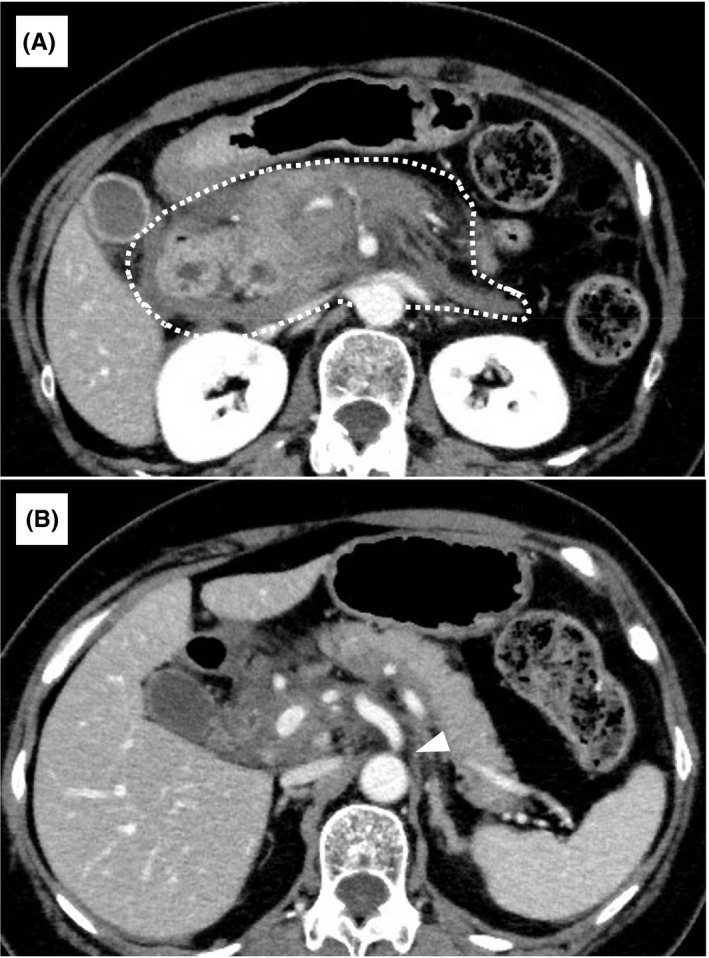
Contrast‐enhanced computed tomography of the abdomen on admission. A, A massive retroperitoneal hematoma below the pancreas and surrounding the pancreas (white dotted line). B, Celiac artery narrowing (white arrowhead)

A 56‐year‐old Japanese woman, with chronic upper abdominal pain and discomfort, consulted our emergency department for sudden epigastric pain. One day prior to her presentation, she developed mild epigastric pain, which improved spontaneously. Two hours before presentation, the pain recurred suddenly after defecation. The pain was severe and waxed and waned in severity. She also reported sweating. She denied having chest pain, back pain, nausea/vomiting, diarrhea, melena, and hematochezia. She had been treated for 5 years for chronic upper abdominal pain, without any findings in upper endoscopy. Her medical history included well‐controlled hyperlipidemia. She had no history of recent trauma. Her medication included omeprazole, domperidone, and rosuvastatin. She occasionally consumed alcohol and had never smoked cigarettes. On admission, her blood pressure was 115/74 mm Hg, heart rate was 63 beats/min, respiratory rate was 28 breaths/min, and the temperature was 35.4°C. She was in acute distress with severe pain. Physical examination revealed epigastric tenderness, without peritoneal signs. Laboratory data revealed leukocytosis, elevated liver enzyme levels, and hyperlactatemia. Results of laboratory examination and arterial blood gas analysis on admission are presented in Table 1. Abdominal contrast‐enhanced computed tomography (CT) revealed retroperitoneal hemorrhage surrounding the duodenum and pancreas (Figure 1A) and celiac artery narrowing (Figure 1B).

**TABLE 1:** Laboratory data

Variables	On admission	The 2nd day of admission	The 10th day of admission
Blood			
White blood cell count (per mm^3^)	14 200	10 000	5500
Hemoglobin (g/dL)	14.1	9.6	10.7
Platelet (per mm^3^)	319 000	215 000	402 000
Alkaline phosphatase (IU/L)	420	263	240
Aspartate transaminase (IU/L)	792	172	26
Alanine transaminase (IU/L)	682	356	31
Lactate dehydrogenase (IU/L)	743	156	366
Total bilirubin (mg/dL)	1.4	0.7	0.8
Amylase (IU/L)	46		
Arterial blood gas (ambient air)			
pH	7.623	7.410	
pCO_2_ (mm Hg)	14.4	36.0	
pO_2_ (mm Hg)	138.7	82.1	
Bicarbonate (mmol/L)	14.6	22.4	
Lactate (mmol/L)	3.29	0.50	

Abbreviations: pCO_2_, partial pressure of carbon dioxide; pO_2_, partial pressure of oxygen.

## 3 DIFFERENTIAL DIAGNOSIS, INVESTIGATIONS, AND TREATMENT

**FIGURE 2:**
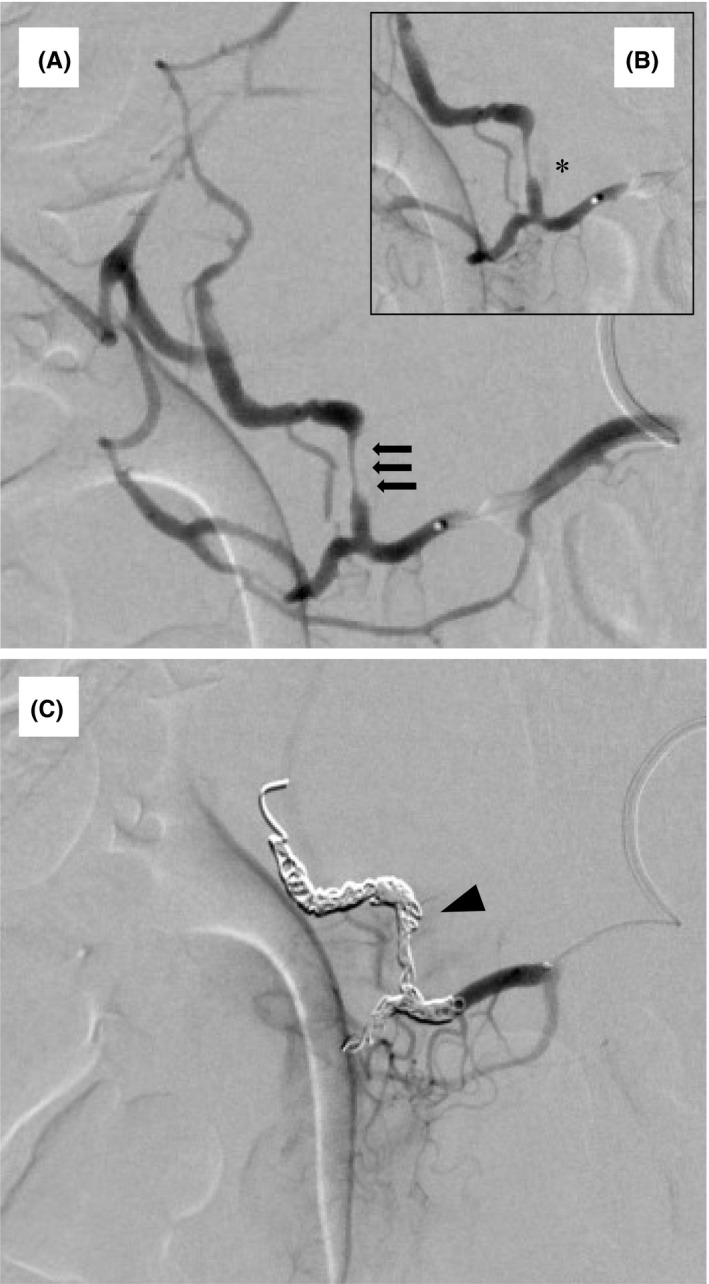
Superior mesenteric artery angiogram. A, Regional narrowing of the posterior inferior pancreaticoduodenal artery (PIPDA) suggestive of dissection (black arrow). B, Extravascular outflow of contrast medium was observed from the PIPDA lesion (black asterixis). C, Coils placed at the distal and proximal segments of PIPDA (black arrowhead)

Based on the abovementioned findings, we suspected that an aneurysm in the celiac artery or superior mesenteric artery (SMA) region was the cause of the retroperitoneal hemorrhage. However, although celiac and SMA angiography revealed no aneurysm, SMA angiography showed regional PIPDA narrowing (Figure 2A). After applying contrast, extravascular outflow was observed from the PIPDA lesion, which was considered a dissection (Figure 2B). Coils were placed at the distal and proximal segments of the PIPDA (Figure 2C). Complete occlusion of the PIPDA was confirmed by resolving the contrast leakage from the same site.

## 4 OUTCOME AND FOLLOW‐UP

Following the intervention, the patient's hemodynamics became stable throughout the hospitalization. Laboratory data, including elevated liver enzyme levels, also improved. She was discharged on hospitalization day 17 and was observed as an outpatient for 15 months, with no recurrence of severe abdominal pain or bleeding.

## 5 DISCUSSION

**FIGURE 3:**
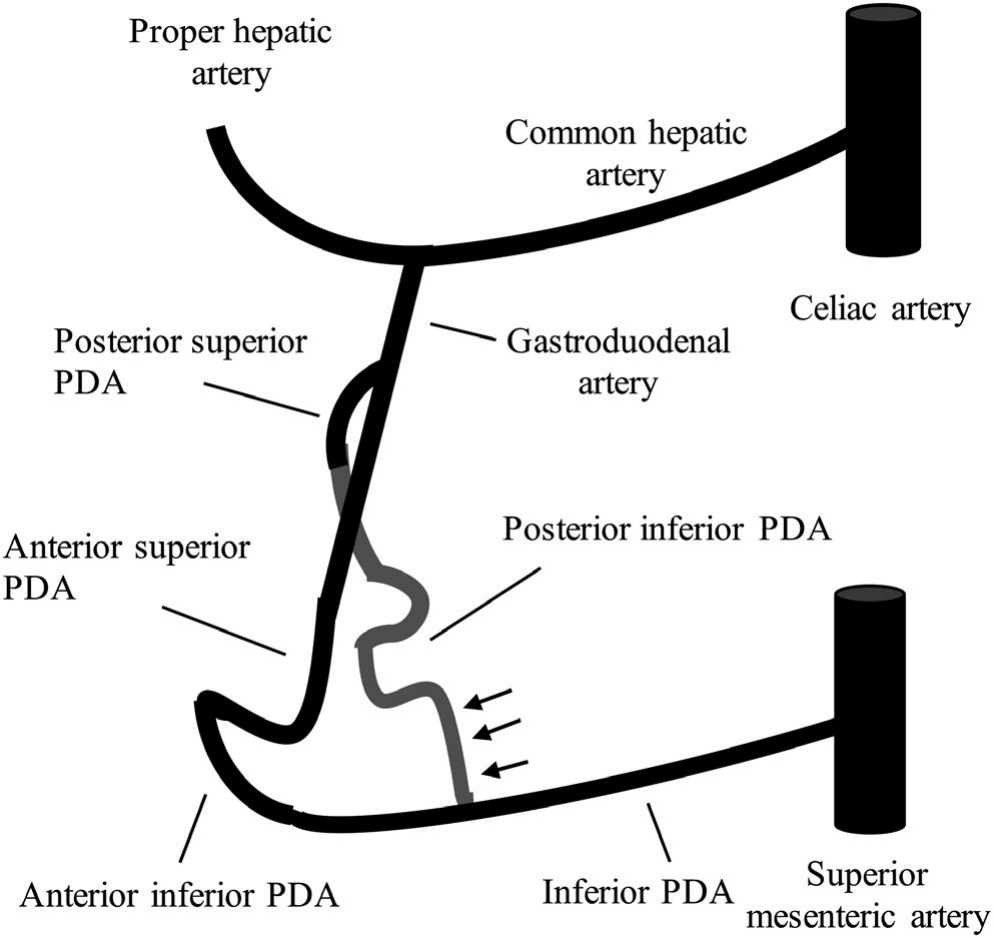
Pancreaticoduodenal arcades and posterior inferior pancreaticoduodenal dissection. The superior and inferior portions of the anterior and posterior pancreaticoduodenal arteries form a pancreatic arcade between the celiac artery and superior mesenteric artery (SMA). Celiac artery stenosis reduces blood flow to the pancreatic arcades, which increases blood flow and pressure in the IPDA from SMA, resulting with PIPDA dissection (black arrows) formation in this case

Retroperitoneal bleeding is a rare but potentially life‐threatening cause of abdominal pain. It is caused by a complication of femoral artery catheterization or other imaging procedures, pelvic/lumber trauma, oral coagulants, and aortic dissection.^5^ Visceral aneurysm and dissection are the rare causes of retroperitoneal bleeding. The IPDA contributes to only 2% of visceral aneurysms.^2^ The mortality rate for ruptured IPDA aneurysms is approximately 30%, requiring early identification and treatment.^4^ ^,^ ^6^ Bleeding from the IPDA dissection has also been reported.^2^ The IPDA aneurysms are associated with celiac artery stenosis, atherosclerosis, infection, trauma, and pancreatitis.^7^ They were related to celiac artery stenosis in 60%‐75% of cases.^8^ Celiac artery stenosis can be detected by contrast‐enhanced CT or Doppler ultrasonography. Since celiac artery stenosis is found in approximately 7% of asymptomatic patients, the presence of typical symptoms, such as chronic abdominal pain (especially postprandial), nausea/vomiting, and mild weight loss, is needed for the diagnosis of CACS.^9^, ^10^ The mechanism by which the celiac artery stenosis leads to the development of IPDA aneurysms is associated with the formation of arterial pancreatic arcade.^11^, ^12^ The superior and inferior portions of the anterior and posterior pancreaticoduodenal arteries form a pancreatic arcade between the celiac artery and SMA. Celiac artery stenosis reduces blood flow to the pancreatic arcades, which increases blood flow and pressure in the IPDA from SMA, promoting the IPDA aneurysm or dissection formation (Figure 3).

In this case, the patient presented with severe abdominal pain, which led to the diagnosis of retroperitoneal hemorrhage. The patient had a celiac artery stenosis detected in CT scan, which, we believe, was the cause of the PIPDA dissection. In addition, the patient had chronic, nonspecific abdominal pain, which may be a symptom of CACS. Typical symptoms of CACS are chronic abdominal pain (especially postprandial), nausea/vomiting, and mild weight loss. In the present case, nausea, vomiting, and weight loss, which are typical symptoms of CACS, were not observed. The other possibility is that atherosclerosis may have caused the PIPDA dissection, not related to CACS, since dyslipidemia is an underlying disease in this case.

The patient had elevated liver enzyme levels, which resolved with treatment; however, the relationship between elevated liver enzyme levels and CACS or IPDA aneurysm/dissection has not been reported in previous literature. Our hypothesis is that blood flow to the liver originating from the celiac artery was reduced due to celiac artery stenosis prior to this episode, and as a result, blood flow from the SMA region to the liver was supplemented via the pancreatic cascade. In this patient, we believe that the PIPDA dissection reduced the total blood flow to the liver from the SMA region, causing transient liver ischemia.

The first‐line treatment for ruptured IPDA aneurysms and dissections is endovascular embolization.^12^ Metallic coils are increasingly used to occlude the ruptured aneurysm or dissection. Regarding embolization, coils are placed at both afferent and efferent arteries close to the bleeding site because of the presence of collateral arteries around the pancreas.^13^, ^14^ In some cases, surgery may be an option due to the complexity of the pancreatic arcade and the difficulty of catheterization due to celiac artery stenosis. Surgical treatment is also indicated when embolization is unsuccessful. Performing surgical ligament release in CACS patients with ruptured aneurysms is not yet established.^3^, ^15^, ^16^ Abdominal artery release surgery is expected to improve the symptoms of CACS and prevent subsequent aneurysms, but its long‐term effects are unknown. In this case, after discussing the benefits and risks of surgery with the patient, we chose to observe the patient without performing surgery.

In conclusion, we report the successful endovascular treatment of ruptured PIPDA dissection associated with CACS. Celiac artery stenosis can cause retroperitoneal hemorrhage secondary to visceral aneurysm and dissection. Clinicians may consider CACS as the cause of visceral aneurysm and dissection in patients with chronic abdominal symptoms.

## CONFLICTS OF INTEREST

None declared.

## AUTHOR CONTRIBUTIONS

KN: drafted the manuscript. HA, TI, JK, and HK: critically revised the manuscript. All authors read and approved the final manuscript.

## ETHICAL APPROVAL

Applicable.

## INFORMED CONSENT

Informed consent was obtained from the patient.

## Data Availability

The data that support the findings of this case report are available from the corresponding author, KN, upon reasonable request.
